# Tropisetron, an Antiemetic Drug, Exerts an Anti‐Epileptic Effect Through the Activation of α7nAChRs in a Rat Model of Temporal Lobe Epilepsy

**DOI:** 10.1111/cns.70086

**Published:** 2024-10-24

**Authors:** Xu Qian, Xinwen Sheng, Jiqiang Ding, Zulipiya Yiming, Jingjun Zheng, Jiagui Zhong, Tengyue Zhang, Xuemei Li, Shuqiao He, Wei Li, Mei Zhang

**Affiliations:** ^1^ Department of Clinical Pharmacy, School of Pharmaceutical Guangzhou Medical University and Key Laboratory of Molecular Target &Clinical Pharmacology Guangzhou China; ^2^ Department of Pharmacy The First Affiliated Hospital of Guangzhou Medical University Guangzhou China; ^3^ Department of Neurosurgery, The Six Affiliated Hospital (Dongguan Eastern Central Hospital) Jinan University Dongguan China

**Keywords:** cognitive impairment, hippocampal sclerosis, neuroinflammation, RNA methylation, synaptic plasticity, temporal lobe epilepsy, Tropisetron, α7nAChRs

## Abstract

**Background:**

Temporal lobe epilepsy (TLE), a prevalent chronic neurological disorder, affects millions of individuals and is often resistant to anti‐epileptic drugs. Increasing evidence has shown that acetylcholine (ACh) and cholinergic neurotransmission play a role in the pathophysiology of epilepsy. Tropisetron, an antiemetic drug used for chemotherapy in clinic, has displayed potential in the treatment of Alzheimer's disease, depression, and schizophrenia in animal models. However, as a partial agonist of α7 nicotinic acetylcholine receptors (α7nAChRs), whether tropisetron possesses the therapeutic potential for TLE has not yet been determined.

**Methods:**

In this study, tropisetron was intraperitoneally injected into pilocarpine‐induced epileptic rats for 3 weeks. Alpha‐bungarotoxin (α‐bgt), a specific α7nAChR antagonist, was applied to investigate the mechanism of tropisetron. Rats were assessed for spontaneous recurrent seizures (SRS) and cognitive function using video surveillance and Morris's water maze testing. Hippocampal impairment and synaptic structure were evaluated by Nissl staining, immunohistochemistry, and Golgi staining. Additionally, the levels of glutamate, γ‐aminobutyric acid (GABA), ACh, α7nAChRs, neuroinflammatory cytokines, glucocorticoids and their receptors, as well as synapse‐associated protein (F‐actin, cofilin‐1) were quantified.

**Results:**

The results showed that tropisetron significantly reduced SRS, improved cognitive function, alleviated hippocampal sclerosis, and concurrently suppressed synaptic remodeling and the m^6^A modification of cofilin‐1 in TLE rats. Furthermore, tropisetron lowered glutamate levels without affecting GABA levels, reduced neuroinflammation, and increased ACh levels and α7nAChR expression in the hippocampi of TLE rats. The effects of tropisetron treatment were counteracted by α‐bgt.

**Conclusion:**

In summary, these findings indicate that tropisetron exhibits an anti‐epileptic effect and provides neuroprotection in TLE rats through the activation of α7nAChRs. The potential mechanism may involve the reduction of glutamate levels, enhancement of cholinergic transmission, and suppression of synaptic remodeling. Consequently, the present study not only highlights the potential of tropisetron as an anti‐epileptic drug but also identifies α7nAChRs as a promising therapeutic target for the treatment of TLE.

## Graphical abstract

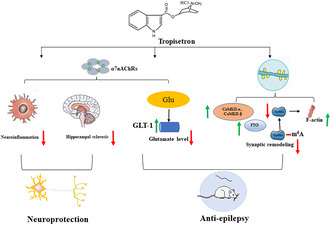

## 1 Introduction

Epilepsy is a chronic neurological disorder that affects over 70 million people worldwide [1]. Among the various types of epilepsy, temporal lobe epilepsy (TLE) stands out as the most prevalent refractory focal epilepsy in adults [2]. In TLE, the primary histological and neuropathological change observed is hippocampal sclerosis (HS) [3]. Despite the introduction of more than 20 new anti‐epileptic medicines (AEMs), some patients still cannot escape the distress of epileptic seizures and have to bear the side effects of AEMs [4]. Consequently, there remains a necessity to pursue the exploration of new targets and effective drugs for the treatment of TLE.

The current comprehension of the neurobiology of TLE indicates that it is characterized by changes in neuronal excitability and synaptic strength [5], leading to synchronized firing in the brain. The interplay between excitatory and inhibitory networks, specifically the glutamatergic‐GABAergic system, plays a crucial role in the initiation and spread of seizures [6]. Recent studies have highlighted the involvement of acetylcholine (ACh) and its interaction with neuronal nicotinic acetylcholine receptors (nAChRs) in regulating neuronal excitability [7]. In hippocampus, the α7 subtype of nAChRs is predominantly expressed [8]. Activation of α7nAChRs has been shown to modulate glutamatergic activity and transmission, leading to excitatory response in hippocampal neurons [9, 10]. Furthermore, α7nAChRs have been implicated in idiopathic generalized epilepsy [11], further supporting their role in epileptogenesis. These findings suggest that α7nAChRs may serve as a potential therapeutic target for the treatment of TLE.

Tropisetron (C_17_H_20_N_2_O_2_), a potent antagonist of 5‐hydroxytryptamine type 3 (5‐HT_3_) receptor and a partial agonist of α7nAChRs, has been used as an antiemetic drug for chemotherapy‐induced nausea and vomiting [12, 13]. Increasing evidences suggested that tropisetron may offer potential treatment benefits for neurological diseases such as Alzheimer's disease, depression, and schizophrenia, through the activation of α7nAChRs [14, 15, 16, 17]. However, the potential of tropisetron to treat TLE remains unknown. Therefore, this study aims to investigate the therapeutic effect of tropisetron in TLE.

## 2 Materials and Methods

### 2.1 Experimental Procedures

#### 2.1.1 Animals

Male Sprague–Dawley rats (200 ± 20 g) were obtained from the Experimental Animal Center of Guangzhou University of Chinese Medicine (SCXK YUE 2013‐0034). The rats were housed under standard laboratory conditions with a 12‐h light/dark cycle and provided with free access to water and food. All experimental procedures were conducted in accordance with the National Institutes of Health Guidelines for Animal Research and were approved by the Ethics Committee for Animal Research of Guangzhou Medical University (Approved No. GY2017‐049).

#### 2.1.2 The Pilocarpine‐Induced Epileptic Rat Model

The pilocarpine‐induced epileptic rat model was established according to our previous research [18]. Rats were intraperitoneally given lithium chloride (127 mg/kg, Sigma‐Aldrich, St. Louis, MO, USA), followed by pilocarpine hydrochloride (30 mg/kg, Sigma‐Aldrich) 18–20 h later. Atropine (1 mg/kg, King York, China) was used to reduce the peripheral cholinergic effects before pilocarpine injection. The evoked seizures were graded according to Racine's scale. Only rats reaching stage IV or V were included in our study. Diazepam (10 mg·kg^−1^, KingYork) was administered to terminate seizures 60 min after the onset of status epilepticus (SE). Survived animals were utilized in the subsequent experiment.

#### 2.1.3 Animal Grouping and Drug Treatment

The rats were divided into experimental and control groups, with each group comprising of 10 rats. Experimental rats were further subdivided into four groups: epilepsy group, Tropisetron + epilepsy group, α‐bgt + tropisetron + epilepsy group, and α‐bgt + epilepsy group. Control rats were randomly assigned to two groups: normal group and α‐bgt + normal group. To investigate the mechanism of tropisetron's action, the α7nAChRs specific blocker, α‐bgt, was utilized. Given its biotoxicity, α‐bgt was administered alone to assess potential adverse reactions in normal rats. Tropisetron (Qilu, China) and α‐bgt (Cayman, USA) were dissolved in saline. Tropisetron was administered daily via intraperitoneal injection at a dose of 3 mg/kg/day for 3 weeks following the onset of spontaneous recurrent seizures (SRS), while α‐bgt was injected intraperitoneally at a dose of 1 μg/kg/day, 30 min prior to tropisetron administration. Rats in the control group received saline injections. All 10 rats underwent video monitoring and the Morris water maze test. Of these, three rats were utilized for Nissl staining and immunohistochemistry analysis, while another three rats were applied for Golgi staining. The remaining four rats were allocated for the measurement of neurotransmitters and the western blotting assay.

### 2.2 Monitoring of Spontaneous Recurrent Seizures

The rats were continuously monitored via video recording in transparent cages utilizing a four‐camera system (Hikvision, China). The frequency, severity, and duration of spontaneous seizures were blindly evaluated by an observer, with seizures classified as Racine level IV or higher being documented. To prevent bias, confirmation of seizure‐like activities in rats was sought from a separate observer.

### 2.3 Morris Water Maze Test

Morris water maze tests were conducted to assess learning and memory. In the acquisition phase, each rat underwent four trials per day for five consecutive days, during which rats were allowed to swim to locate the submerged platform. Path length, escape latency (time taken to reach the platform), and mean swimming speed were recorded using a computerized video tracking system. Following the 5‐day acquisition training period, a spatial probe trial was administered. The time spent in the target quadrant (measured as target crossing) was calculated within a 120 s.

### 2.4 Nissl Staining, Immunofluorescence Staining, and Immunohistochemistry

Under 1.5% isoflurane anesthetized, a catheter was inserted into the ascending aorta, and rats were perfused with 4% paraformaldehyde (PFA). The brains were removed and postfixed in 4% PFA overnight at 4°C. Subsequently, the brains underwent dehydration, transparency enhancement, and paraffin embedding. Serial coronal sections containing the hippocampus were cut and collected sequentially.

Nissl staining was utilized to assess hippocampal formation, involving deparaffinization, rehydration, and staining with 1% toluidine blue. Neurons in the cell layer were measured and quantified using the ImageJ/NIH image analysis system. For quantitative analysis, every 15th staining section was selected (five sections per rat). Images of CA1, CA3, and hilus of hippocampus sections were captured at 40× magnification.

Immunohistochemical and immunofluorescence analyses were conducted following to standard protocol. For the immunohistochemistry procedure, the hippocampal sections were incubated overnight at 4°C with an anti‐α7nAChR antibody (0.5 mg/mL, Abcam). After washes, the sections were incubated with a biotinylated anti‐rabbit IgG (1:1000, Abcam) for 1 h at 37°C, followed by development via DAB reagent. Images of the CA1 and CA3 regions were captured at 90× magnification, and positively labeled neurons were quantified using ImageJ software. For immunofluorescence analysis, the treated hippocampal sections were incubated overnight with primary antibodies (anti‐α7nAChRs mouse mAb, 1:100, CST; anti‐NeuN rabbit mAb, 1:200, MERCK; and anti‐GFAP goat mAb, 1:200, CST) at 4°C. After washing, the slices were incubated with IgG‐alexa fluor‐488 (goat anti‐mouse, 1:200, Abkine), IgG‐alexa fluor‐549 (goat anti‐rabbit, 1:200, Abkine), and IgG‐alexa fluor‐647 (donkey anti‐goat, 1:200, Abkine) in the dark for 1 h. Subsequently, the DAPI dye solution (CST) was used to stain the nucleus for 10 min. The slices were observed with a confocal microscope, and images of the coverslip were obtained at 60× magnification.

### 2.5 Golgi Staining

Golgi staining was utilized to examine the structure of dendritic spines in accordance with our previous research [19]. The brains were fixed, embedded, and hippocampal sections were sliced into 100 μm thickness, then mounted with glycerin gelatin. Images of the hippocampal sections were captured using a digital slice scanner. Dendritic segments, each 25 μm in length, from 10 neurons per animal were chosen in the dentate gyrus region for counting the numbers of dendritic spines. Dendritic spine densities were quantified as the number of spines per unit length.

### 2.6 Measurement of Neurotransmitters

Hippocampal tissues were homogenized with ice‐cold phosphate‐buffered saline and centrifuged at 12,000 rpm for 10 min at 4°C to obtain supernatants. Glutamate and acetylcholine (ACh) levels were quantified using colorimetry following the manufacturer's instructions (Nanjing Jian Cheng Bioengineering Institute, China). The content of γ‐aminobutyric acid (GABA) was determined using commercial ELISA kits according to the manufacturer's instructions (Cloud‐Clone Corp, USA). Absorbance was measured at 450 nm using a microplate reader.

### 2.7 Measurement of Inflammatory Cytokines and Glucocorticoids

The levels of TNF‐α (Neobioscience, China), IL‐1β (Neobioscience, China), and corticosterone (R&D Systems, USA) in serum were quantified using commercial ELISA kits following the manufacturer's protocols. Absorbance was measured at 450 nm using a microplate reader.

### 2.8 RNA Isolation and Quantitative Real‐Time PCR

RNA was extracted using Trizol (Life Technologies) according to the manufacturer's protocol. cDNA was synthesized using the transcript All‐in‐One First‐Strand cDNA Synthesis SuperMix for Quantitative Polymerase Chain Reaction (qPCR) (Takara). Quantitative real‐time Polymerase Chain Reaction (PCR) was carried out using Fast SYBR Green PCR Master Mix (Applied Biosystems) on a Step‐One Fast Real‐time PCR System (Applied Biosystems). Data analysis was performed using the $2^{-\Delta \Delta C_{T}}$ method. The primers used in this study are listed below.

**Table:** 

Cofilin‐1 Forward primer	ACTGACAGGAATCAAGCACGA
Cofilin‐1 Reverse primer	TTGCCCTCCAGGGAAATGAC
GAPDH Forward primer	AGTGCCAGCCTCGTCTCATA
GAPDH Reverse primer	TTGAACTTGCCGTGGGTAGA

### 2.9 Methylated RNA Immunoprecipitation (MeRIP) Analysis

Total cellular RNA was extracted from fresh rat hippocampi using the Trizol reagent following the manufacturer's instructions (Life Technologies). The RNA was then fragmented into 100–200 bp pieces. Next, 600 ng of RNA fragments underwent m^6^A‐SMART‐seq using anti‐m^6^A rabbit polyclonal antibody (Synaptic Systems, 202003) as previously described. Briefly, 5 μg of anti‐m^6^A polyclonal antibody was conjugated to Dynabeads Protein A (Thermo Fisher; 10001D) and utilized for each affinity pull‐down. The m^6^A RNA was eluted twice with 6.7 mM N^6^‐Methyladenosine (Sigma‐Aldrich; M2780) in 1 × IP buffer (10 mM Tris–HCl pH 7.5, 150 mM NaCl, and 0.1% (vol/vol) Igepal CA‐630) and purified using RNA Clean and Concentrator‐5 (Zymo Research). Subsequently, the m^6^A‐modified RNA was analyzed using the quantitative real‐time PCR protocol as described. The primers for cofilin‐1 used in this study are listed below.

**Table:** 

Cofilin‐1 forward primer	GCTAACTGCTACGAGGAGGTCA
Cofilin‐1 reverse primer	CTGGAGGTGGCTCACAAAGG

### 2.10 Western Blot Analysis

The hippocampal samples were separated using 10% SDS‐PAGE and transferred to polyvinylidene fluoride (PVDF) membranes (Millipore). The membranes were probed with anti‐GR (1:1000, Abcam), anti‐GLT‐1, anti‐NMDA, anti‐cofilin‐1, anti‐CaMKII‐α, anti‐CaMKII‐β, anti‐F‐actin, anti‐Mettl3, anti‐Mettl14, and anti‐GFAP (1:1000, CST) and anti‐FTO (1: 1000, Abcam) antibodies at 4°C overnight. After washing with TBST, the membranes were incubated with HRP‐conjugated anti‐rabbit or anti‐mouse secondary antibodies (1:10,000, ZSGB‐BIO) for 1 h at room temperature. Blots were developed using ECL‐Plus (Perkin Elmer) and analyzed with Quantity One software.

### 2.11 Statistical Analyses

In this study, quantitative results were derived from a minimum of three specimens and three independent experiments. The experimental data were analyzed using GraphPad Prism 9.5.1 (GraphPad, San Diego, CA) and are presented as the mean ± standard error of the mean (SEM). Shapiro–Wilk normality test was applied to evaluate the normality of data distribution. Homogeneity of variances among groups was assessed using one‐way analysis of variance (ANOVA). The Bonferroni method was employed for multiple comparisons of means between two groups. Statistical significance was set at *p* < 0.05.

## 3 Results

### 3.1 Tropisetron Exhibits Anti‐Epileptic Effect in TLE Rats

**FIGURE 1:**
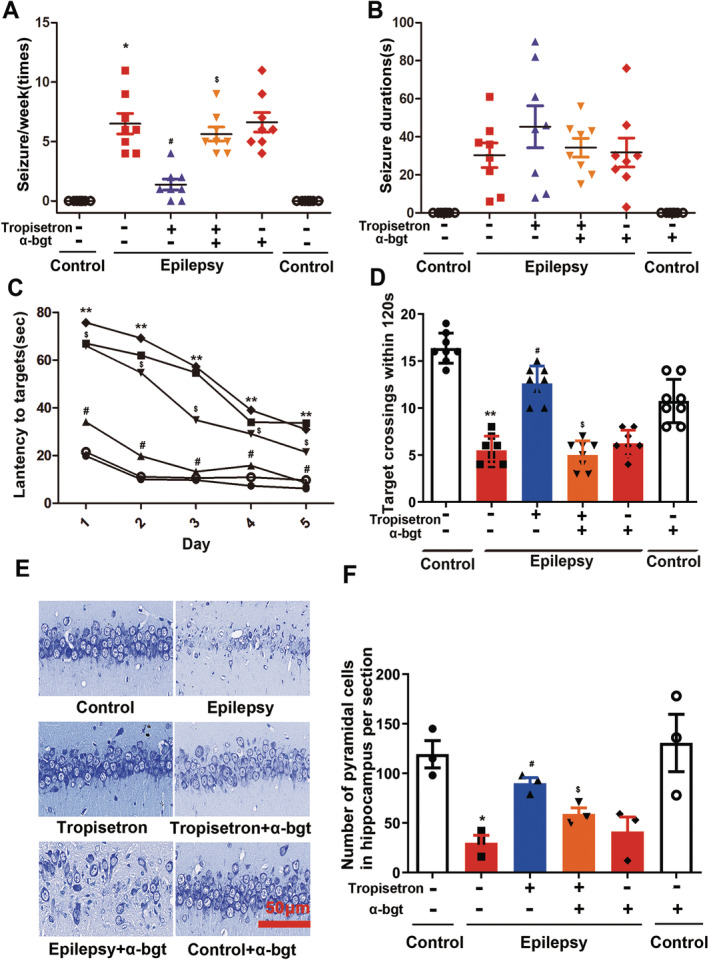
Tropisetron demonstrates a significant anti‐epileptic effect in TLE rats. (A) The numbers and durations of SRS were recorded; (B) The duration of SRS was recorded; *p < 0.05, control versus epilepsy; ^#^ *p* < 0.05, epilepsy versus epilepsy + tropisetron; ^$^ *p* < 0.05, epilepsy + tropisetron versus epilepsy + tropisetron + α‐bgt; Mean ± SEM, *n* = 8. (C) The latency to reach the underwater platform was evaluated using the Morris water maze; (D) the number of crossings over the target quadrant; **p < 0.01, control versus epilepsy; ^#^ *p* < 0.05, epilepsy versus epilepsy + tropisetron; ^$^ *p* < 0.05, epilepsy + tropisetron versus epilepsy + tropisetron + α‐bgt; Mean ± SEM, *n* = 8. (E) Nissl staining was used to assess neuronal damage in the hippocampal regions; (F) Quantitative analysis of pyramidal neurons in the hippocampal regions. *p < 0.05, control versus epilepsy; ^#^ *p* < 0.05, epilepsy versus epilepsy + tropisetron; ^$^ *p* < 0.05, epilepsy + tropisetron versus epilepsy + tropisetron + α‐bgt; Mean ± SEM, *n* = 3.

Tropisetron was used to treat TLE rats for 3 weeks following the establishment of a pilocarpine‐induced epileptic model. In the present study, spontaneous recurrent seizures (SRS) occurred on average 12 days after the induction of status epilepticus in the rats. The administration of tropisetron for 3 weeks notably reduced the frequency of SRS without affecting the duration of seizures (Figure 1A,B). These findings suggest that tropisetron exhibits an anti‐epileptic effect in TLE rats.

Subsequently, the Morris water maze (MWM) was utilized to investigate the impact of tropisetron on TLE‐induced cognitive impairment. During the visible platform training, tropisetron decreased the latency to reach the target platform during the 5‐day training trials, while swimming activity remained unaffected (Figure 1C,D). Furthermore, in the probe test, tropisetron treatment led to an increase in the number of visits to the previous platform location in epileptic rats (Figure 1C,D). These findings suggest that tropisetron treatment effectively ameliorated the cognitive deficits observed in epileptic rats.

Additionally, Nissl staining was used to assess the impact of tropisetron on hippocampal neurons. Our results revealed that pyramidal neurons in the hippocampal regions of normal rats were regularly aligned and displayed intact structures with distinct nucleoli (Figure 1E,F). In contrast, the hippocampal regions of epileptic rats exhibited disrupted layered structures of pyramidal neurons, accompanied by evident neuronal loss, and an irregular arrangement of pyramidal neurons with an atactic shape, opaque cytoplasm, and shrinking nuclei (Figure 1E,F). Tropisetron treatment significantly mitigated the epileptic‐induced loss of pyramidal neurons and ameliorated these pathological changes in the hippocampus (Figure 1E,F).

Importantly, the administration of α‐bgt, an antagonist of α7nAChR, abolished the effect of tropisetron without causing any toxicity's reactions in control rats. This observation strongly suggests the involvement of α7nAChRs in tropisetron's anti‐epileptic effect.

### 3.2 Tropisetron Attenuates Hippocampal Sclerosis in TLE Rats

#### 3.2.1 Tropisetron Ameliorates Hippocampal Neuron Loss in Rats

**FIGURE 2:**
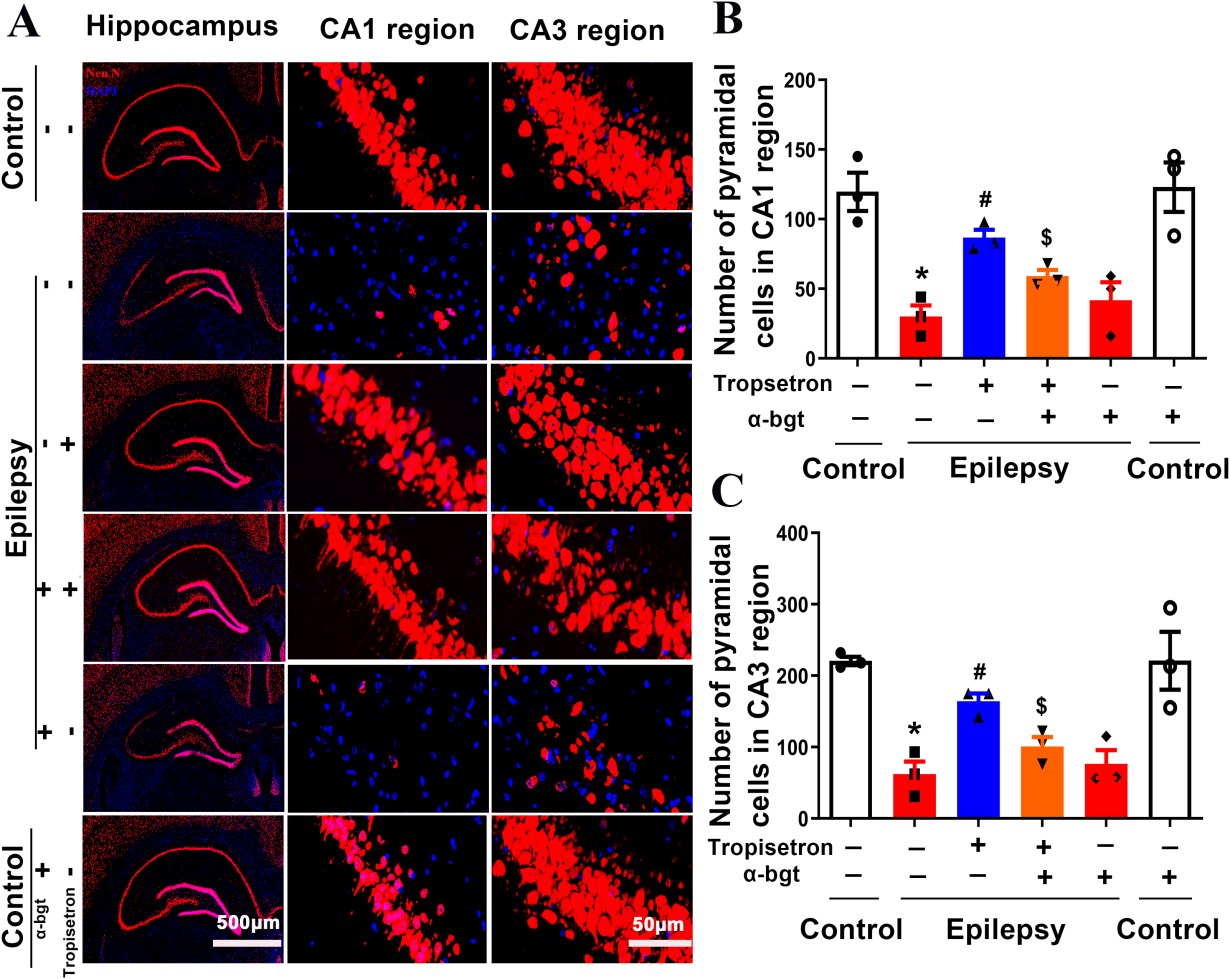
Tropisetron attenuates hippocampal damage in epileptic rats. (A) Immunofluorescence was used to observe the expression of Neu N in hippocampus (DAPI‐ blue, and Neu N‐ red) (5×). CA1 and CA3 regions of the hippocampus (90×). (B) Quantitative analysis of pyramidal neurons in CA1 regions. (C) Quantitative analysis of pyramidal neurons in CA3 regions. *p < 0.05, control versus epilepsy; ^#^ *p* < 0.05, epilepsy versus epilepsy + tropisetron; ^$^ *p* < 0.05, epilepsy + tropisetron versus epilepsy + tropisetron + α‐bgt; Mean ± SEM, *n* = 3.

HS is the neuropathological hallmark of TLE, characterized by the loss of hippocampal neuronal and reactive astrogliosis [3]. In our present study, Neu N‐positive cells with red nuclei are mature neurons, forming a dense layer of granular cells. The number of Neu N‐positive cells per unit area in epileptic rats was significantly less than that of control rats. The CA1 regions exhibited a disruption in the laminated structure of Neu N‐positive cells, with a noticeable loss. Additionally, the CA3 regions displayed a disordered arrangement of Neu N‐positive cells, along with a decrease in the number of Neu N‐positive cells in the hippocampi of epileptic rats (Figure 2A–C). However, tropisetron treatment alleviated the observed pathological changes in both the CA1 and CA3 regions of the hippocampus, as depicted in Figure 2A–C. Importantly, the neuroprotective effect of tropisetron on hippocampal neurons was abolished when α‐bgt was administered, suggesting that tropisetron's action in preserving neuronal integrity is associated with α7nAChRs.

#### 3.2.2 Tropisetron Suppresses Astrogliosis in TLE Rats

**FIGURE 3:**
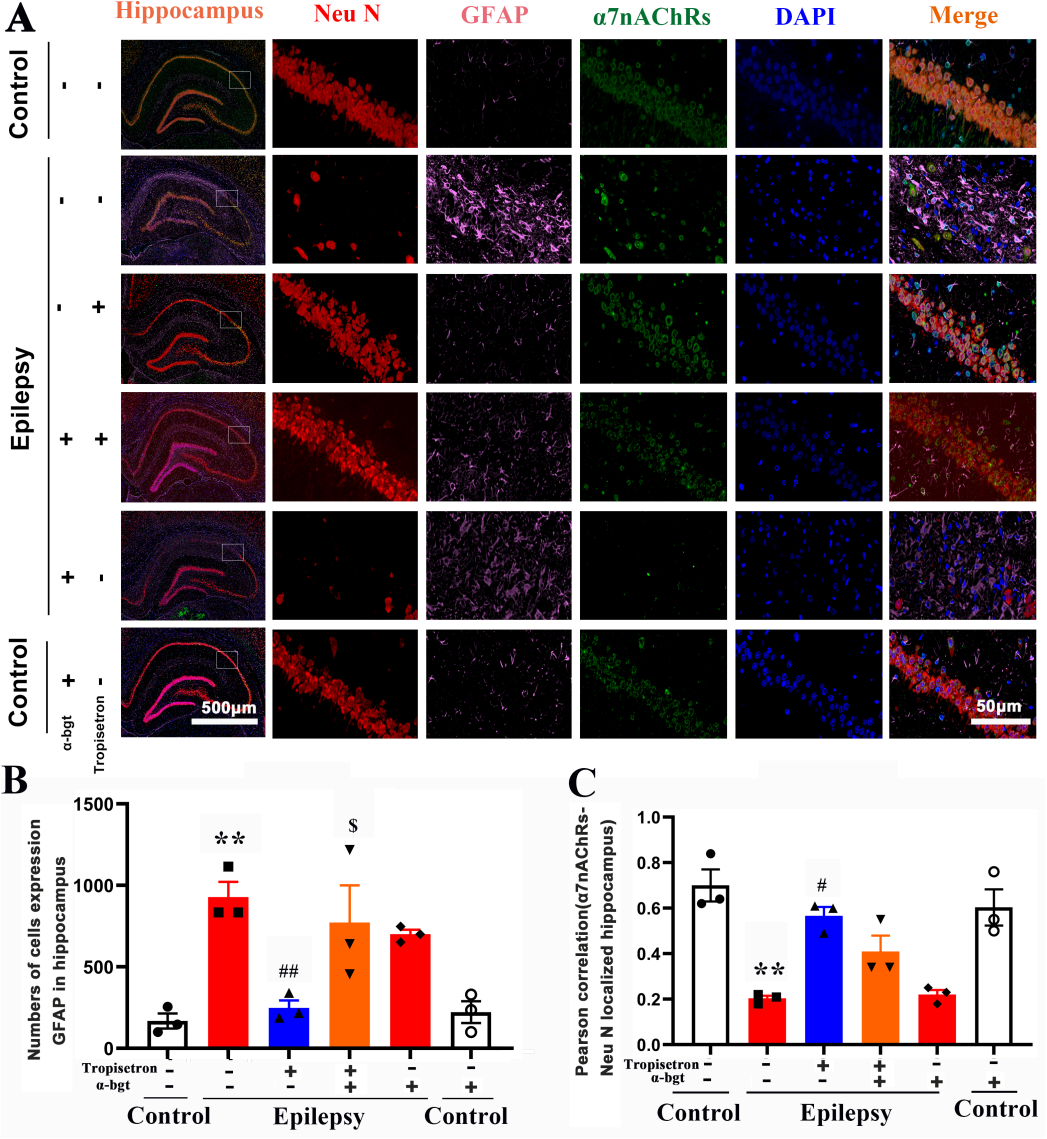
Tropisetron alleviates hippocampal sclerosis in TLE rats via α7nAChRs. (A) The staining of Neu N (red), GFAP (pink), and α7nAChs (green) was performed to observe hippocampal sclerosis in the hippocampus. (B) Quantitative analysis of astrocytes in the hippocampal regions. (C) Quantitative analysis of the colocalization between neuron and α7nAChRs. **p < 0.01, control versus epilepsy; ^#^ *p* < 0.05, epilepsy versus epilepsy + tropisetron; ^##^ *p* < 0.01, epilepsy versus epilepsy + tropisetron; ^$^ *p* < 0.05, epilepsy + tropisetron versus epilepsy + tropisetron + α‐bgt; Mean ± SEM, *n* = 3.

Triple‐label immunofluorescence staining was performed to observe astrogliosis in the hippocampus using Neu N (red), GFAP (pink), and α7nAChs (green). The present results showed an increase in GFAP‐positive cells in the hippocampus of TLE rats (Figure 3A,B), indicating astrogliosis. Furthermore, immunofluorescence analysis revealed a reduction in α7nAChs‐positive cells accompanied by a decrease in Neu N‐positive cells in TLE rats (Figure 3A–C). This observation suggests a co‐localization between Neu N and α7nAChRs (Figure 3A–C). Notably, tropisetron treatment significantly mitigated the increase in GFAP‐positive cells and enhanced the co‐localization of Neu N and α7nAChRs (Figure 3A–C). Intriguingly, α‐bgt antagonized the effect of tropisetron. Therefore, these results suggest that the inhibitory action of tropisetron on HS is associated with the activity of a7nAChRs in neurons.

### 3.3 Tropisetron Reduces Glutamate Level and Enhances Cholinergic Transmission in TLE Rats

**FIGURE 4:**
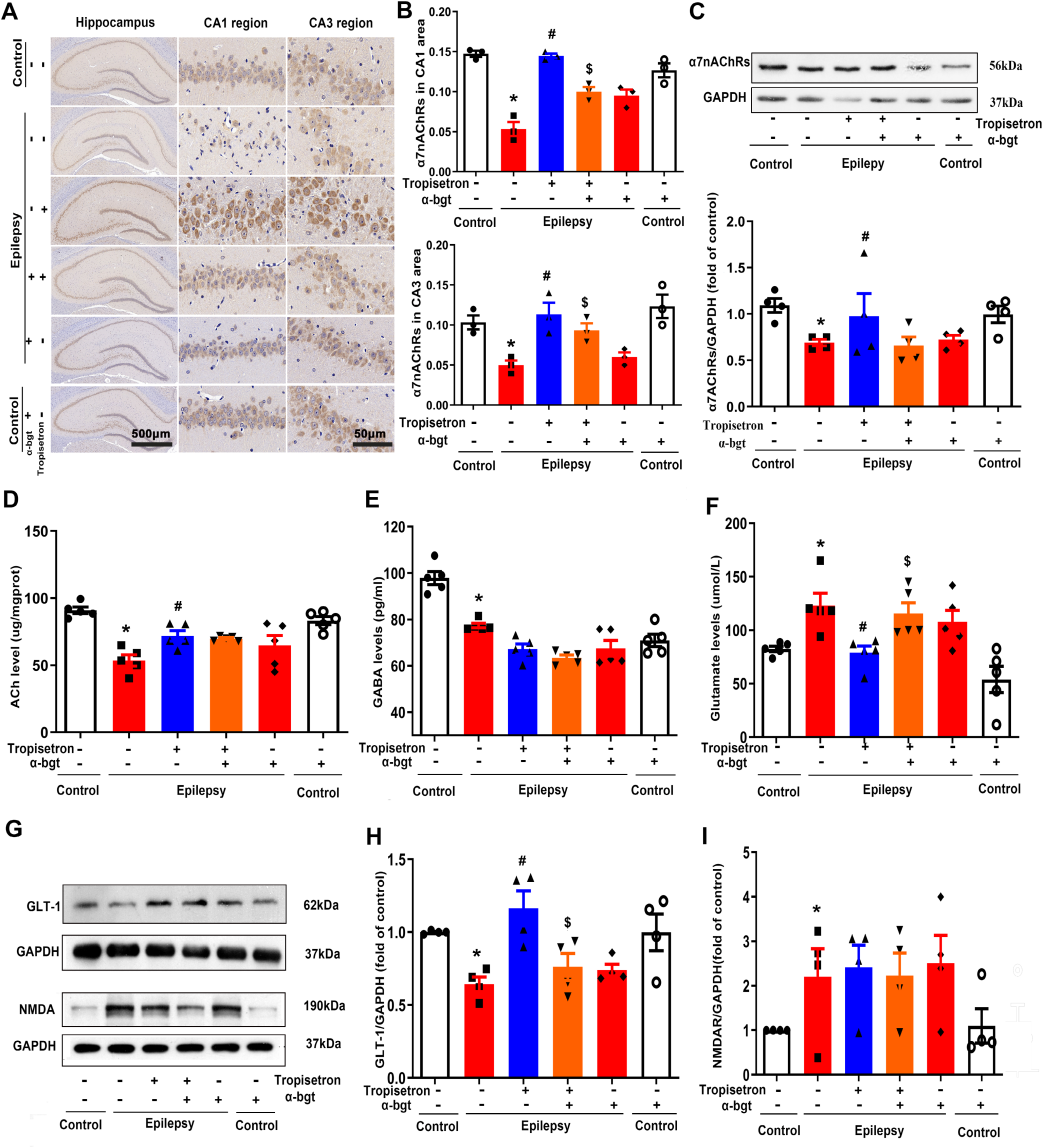
Tropisetron reduces glutamate level and enhances cholinergic transmission in TLE rats. (A) Immunohistochemistry staining was used to visualize the expression of α7nAChRs in hippocampus; (B) Quantitative analysis of α7nAChRs in CA1 and CA3 regions; *p < 0.05, control versus epilepsy; ^#^ *p* < 0.05, epilepsy versus epilepsy + tropisetron; ^$^ *p* < 0.05, epilepsy + tropisetron versus epilepsy + tropisetron + α‐bgt; Mean ± SEM, *n* = 3. (C) The expression of α7nAChRs in hippocampus was stained by western blotting. *p < 0.05, control versus epilepsy; ^#^ *p* < 0.05, epilepsy versus epilepsy + tropisetron; Mean ± SEM, *n* = 4. (D–F) ACh, GABA, and glutamate levels in hippocampus were measured by colorimetry. *p < 0.05, control versus epilepsy; ^#^ *p* < 0.05, epilepsy versus epilepsy + tropisetron; ^$^ *p* < 0.05, epilepsy + tropisetron versus epilepsy + tropisetron + α‐bgt; Mean ± SEM, *n* = 5. (G) The expression of GLT‐1 and NMDA was stained by western blotting. (H) Quantitative analysis of GLT‐1. (I) Quantitative analysis of NMDA; *p < 0.05, control versus epilepsy; ^#^ *p* < 0.05, epilepsy versus epilepsy + tropisetron; ^$^ *p* < 0.05, epilepsy + tropisetron versus epilepsy + tropisetron + α‐bgt; Mean ± SEM, *n* = 4.

Further investigation was conducted to elucidate the therapeutic mechanism of tropisetron in TLE rats. The present results showed that epileptic rats exhibited an increase in glutamate levels and a decrease in GABA levels in the hippocampus (Figure 4E,F), indicating an imbalance in neuronal excitation and inhibition within the TLE brain. However, tropisetron treatment decreased glutamate levels without altering GABA levels (Figure 4E,F). Additionally, a decrease in GLT‐1, a glutamate transporter involved in the reuptake of excessive glutamate from the synaptic cleft [20], was observed in the hippocampus, indicating a disruption of glutamate metabolism in TLE. Notably, tropisetron treatment increased the expression of GLT‐1, indicating tropisetron's involvement in glutamate reuptake. Neuronal hyperexcitation mediated by excessive glutamate occurs through the activation of NMDA receptors [21]. The present study revealed an increase in NMDA receptor expression in epileptic rats. However, tropisetron did not reverse this alteration, suggesting that its inhibition of neuronal hyperexcitation is not directly related to NMDA receptors (Figure 4G–I). Crucially, the effects of tropisetron on glutamate levels and GLT‐1 expression were inhibited by α‐bgt, indicating that tropisetron regulates glutamate metabolism by α7nAChRs during TLE.

ACh and its nAChRs have been shown to regulate neuronal excitability [22]. Our experiments unveiled that the epileptic rats showed the decreased levels of ACh in the hippocampus and reduced expression of α7nAChRs in both hippocampal slices and tissue homogenates (Figure 4A–D), indicating cholinergic impairment in epileptic rats. However, treatment with tropisetron increased ACh levels and α7nAChR expression, demonstrating its ability to enhance cholinergic transmission. It is noteworthy that α‐bgt only blocked the effects of tropisetron on α7nAChR expression, rather than ACh levels (Figure 4A–D). Furthermore, when used alone, α‐bgt tended to increase ACh levels in control rats.

### 3.4 Tropisetron Suppresses Neuroinflammatory Responses in TLE Rats

**FIGURE 5:**
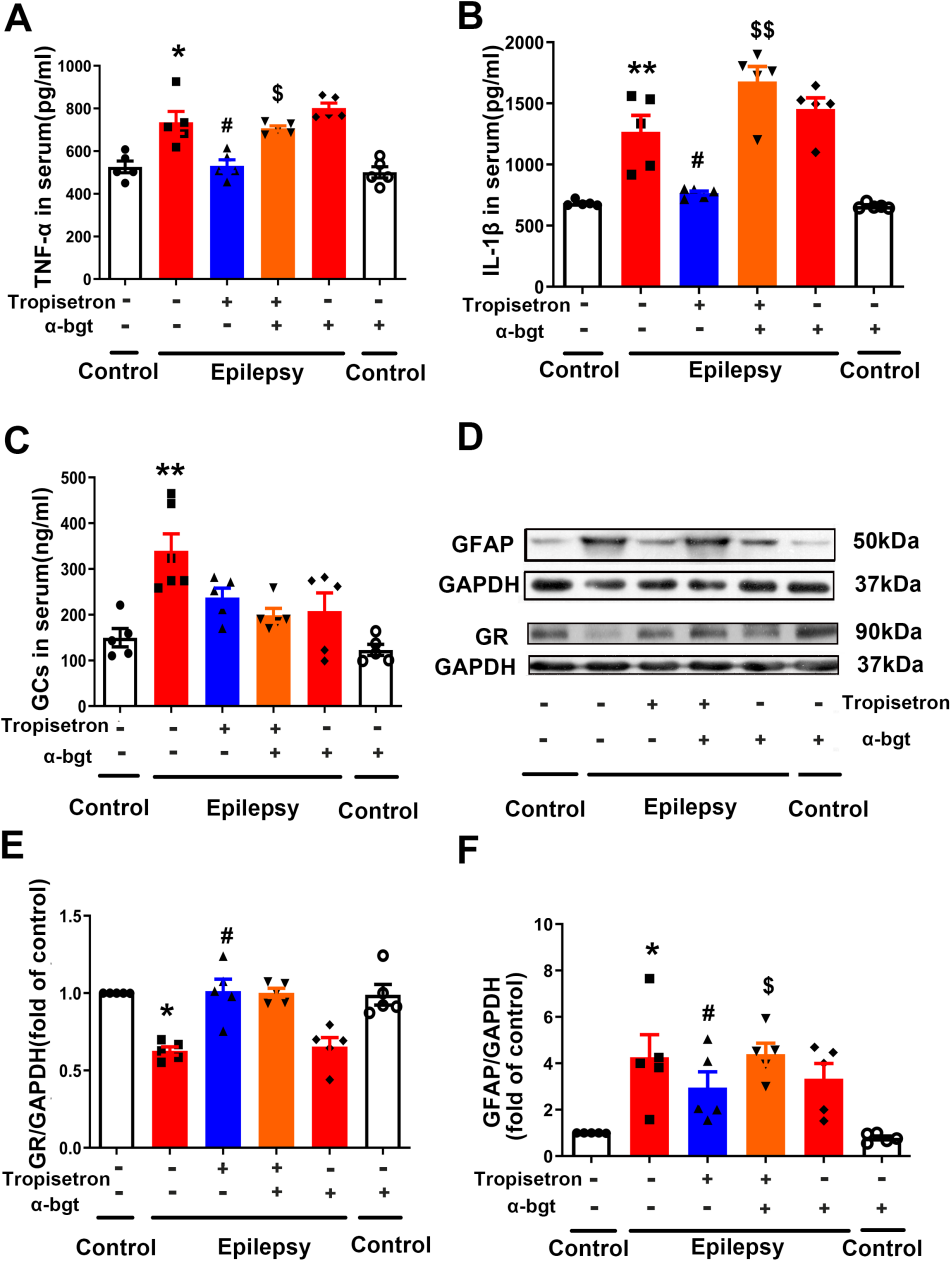
Tropisetron suppresses neuroinflammation in epileptic rats. (A, B) The levels of TNF‐α and IL‐1β in the sera were assessed using ELISA. (C) The corticosterone levels in the sera were assessed using ELISA. (D) The expression of GFAP and GR in the hippocampus was analyzed by western blotting. (E) Quantitative analysis of GR expression. (F) Quantitative analysis of GFAP expression; *p < 0.05, control versus epilepsy; **p < 0.01, control versus epilepsy; ^#^ *p* < 0.05, epilepsy versus epilepsy + tropisetron; ^$^ *p* < 0.05, epilepsy + tropisetron versus epilepsy + tropisetron + α‐bgt; ^$$^ *p* < 0.01, epilepsy + tropisetron versus epilepsy + tropisetron + α‐bgt; Mean ± SEM, *n* = 5.

Both animal models of epilepsy and patients with epilepsy commonly exhibit neuroinflammation, which plays a significant role in the progression of TLE [23]. In this study, it was demonstrated that tropisetron effectively reduced the levels of TNF‐α and IL‐1β in the sera of epileptic rats (Figure 5A,B), indicating its ability to suppress intracerebral inflammation in TLE rats. Additionally, neuroinflammation triggers the activation of astrocytes [24]. The increased expression of glial fibrillary acidic protein (GFAP), a marker of astrocytes' active neuroinflammation state [25], was observed in epileptic rats. However, tropisetron reversed this change (Figure 5D,F), suggesting its potential in reducing neuroinflammation in TLE rats. Importantly, the actions of tropisetron on the above‐mentioned effects were inhibited by α‐bgt. This suggests that α7nAChRs are involved in tropisetron's anti‐inflammatory effect.

Glucocorticoids (GCs) play a role in modulating the intracerebral inflammatory response. Our study demonstrated a significant increase in corticosterone levels and a decline in GR expression in the hippocampus of epileptic rats (Figure 5C). Interestingly, tropisetron only increased GR expression without affecting corticosterone levels in the hippocampus (Figure 5D,E). However, α‐bgt failed to prevent the effects of tropisetron mentioned above.

### 3.5 Tropisetron Inhibits Synaptic Plasticity in the Hippocampi of TLE Rats

**FIGURE 6:**
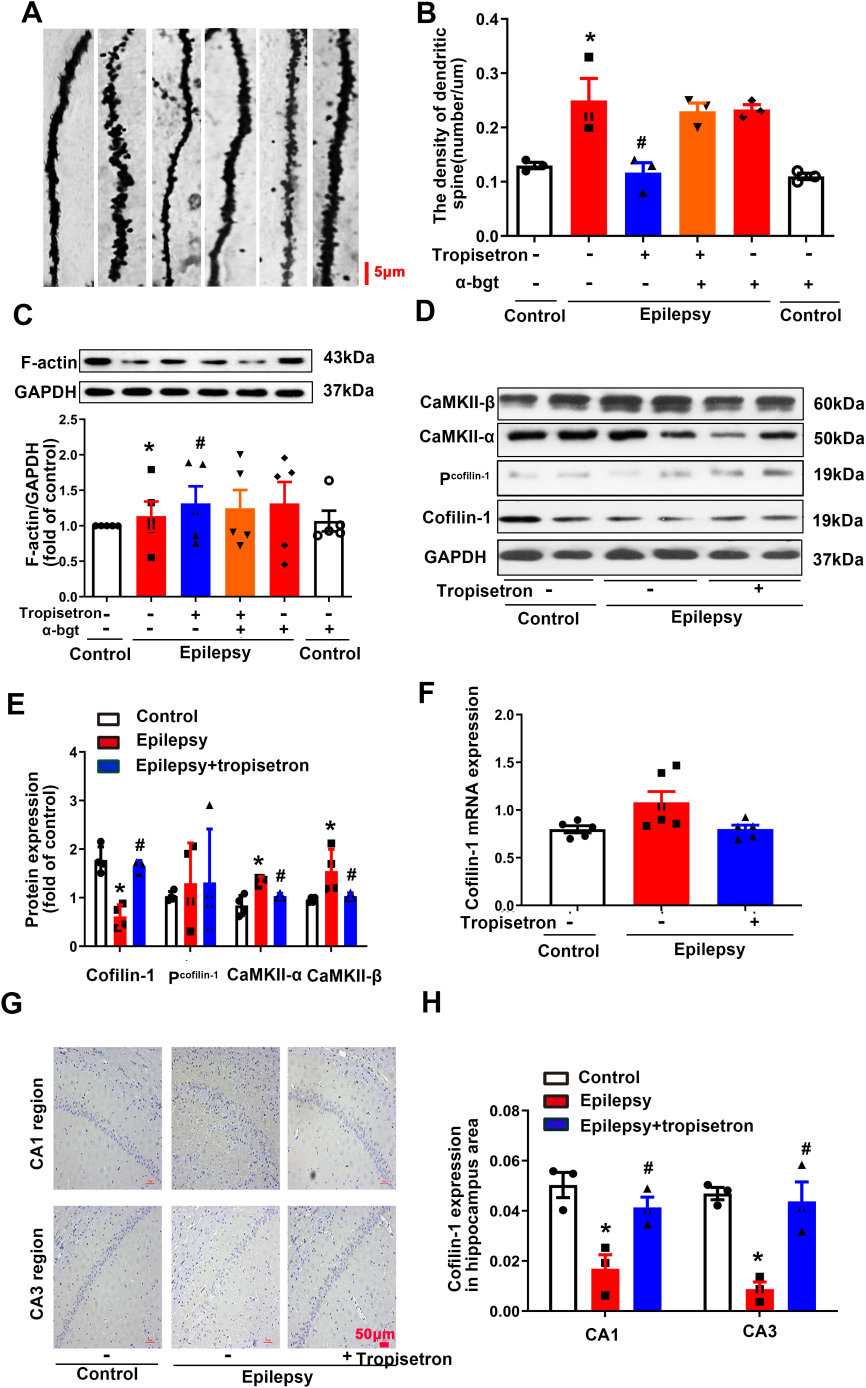
Tropisetron inhibits synaptic remodeling in the hippocampi of epileptic rats. (A) Dendritic spines were dyed by Golgi staining in the dentate gyrus of hippocampus. (B) Quantitative analysis of dendritic spines densities; (C–E) The expression of F‐actin, cofilin‐1, P^cofilin‐1^, CaMKII‐α, and CaMKII‐β proteins in the hippocampus were analyzed by western blotting; (F–H) The expression of cofilin‐1 in hippocampus was stained by immunohistochemistry. *p < 0.05, control versus epilepsy; ^#^ *p* < 0.05, epilepsy versus epilepsy + tropisetron; ^$^ *p* < 0.05, epilepsy + tropisetron versus epilepsy + tropisetron + α‐bgt; Mean ± SEM, *n* = 3.

Synaptic reorganization is implicated in the hippocampal hyperexcitability observed in TLE [26]. The structural modification of dendritic spines has been shown to play a critical role in synaptic plasticity [27]. The density of dendritic spines was found to be significantly increased in epileptic rats compared to control rats (Figure 6A,B), indicating synaptic remodeling in the hippocampus of epileptic rats. However, tropisetron treatment reduced the number of dendritic spines, suggesting that tropisetron inhibits synaptic reorganization. CaMKII plays a structural role in linking functional and structural plasticity in dendritic spines [28]. The expression of CaMKII‐α and CaMKII‐β was found to be elevated in the hippocampus of TLE rats (Figure 6D,E), indicating a dysfunction in CaMKII in TLE rats. However, tropisetron administration restored this process (Figure 6D,E), illustrating that tropisetron modulates CaMKII function in TLE. Interestingly, the effects of tropisetron on synaptic remodeling were not blocked by α‐bgt, suggesting that the inhibitory effect of tropisetron on synaptic reorganization is not associated with α7nAChRs.

F‐actin, the main cytoskeletal protein in dendritic spine, plays a pivotal role in synaptic formation, plasticity, and stability [29]. Reduced expression of F‐actin was observed in the hippocampi of epileptic rats, indicating a disruption in the spine cytoskeleton (Figure 6C). Tropisetron treatment restored this change, suggesting that tropisetron helps maintain synaptic plasticity and stability. Cofilin‐1, an actin‐binding protein, preserves the stability of dendritic spine by regulating F‐actin depolymerization to control its dynamics and function [30]. The present study demonstrated a decrease in cofilin‐1 levels in both hippocampal slices and tissue homogenates from epileptic rats, while the expression of cofilin‐1 mRNA in the hippocampus remained unchanged (Figure 6D–H), suggesting the presence of post‐transcriptional modification of cofilin‐1 in TLE rats. Importantly, tropisetron treatment reversed this alteration (Figure 6D–H), indicating that tropisetron restored its malfunction. It should be mentioned that the phosphorylation levels of cofilin‐1 remained unchanged in epileptic rats, implying that the modifications in the expression of cofilin‐1 are unlikely to be attributed to post‐translational regulatory mechanisms.

### 3.6 Tropisetron Inhibits Cofilin‐1 m^6^A RNA Methylation in TLE Rats

**FIGURE 7:**
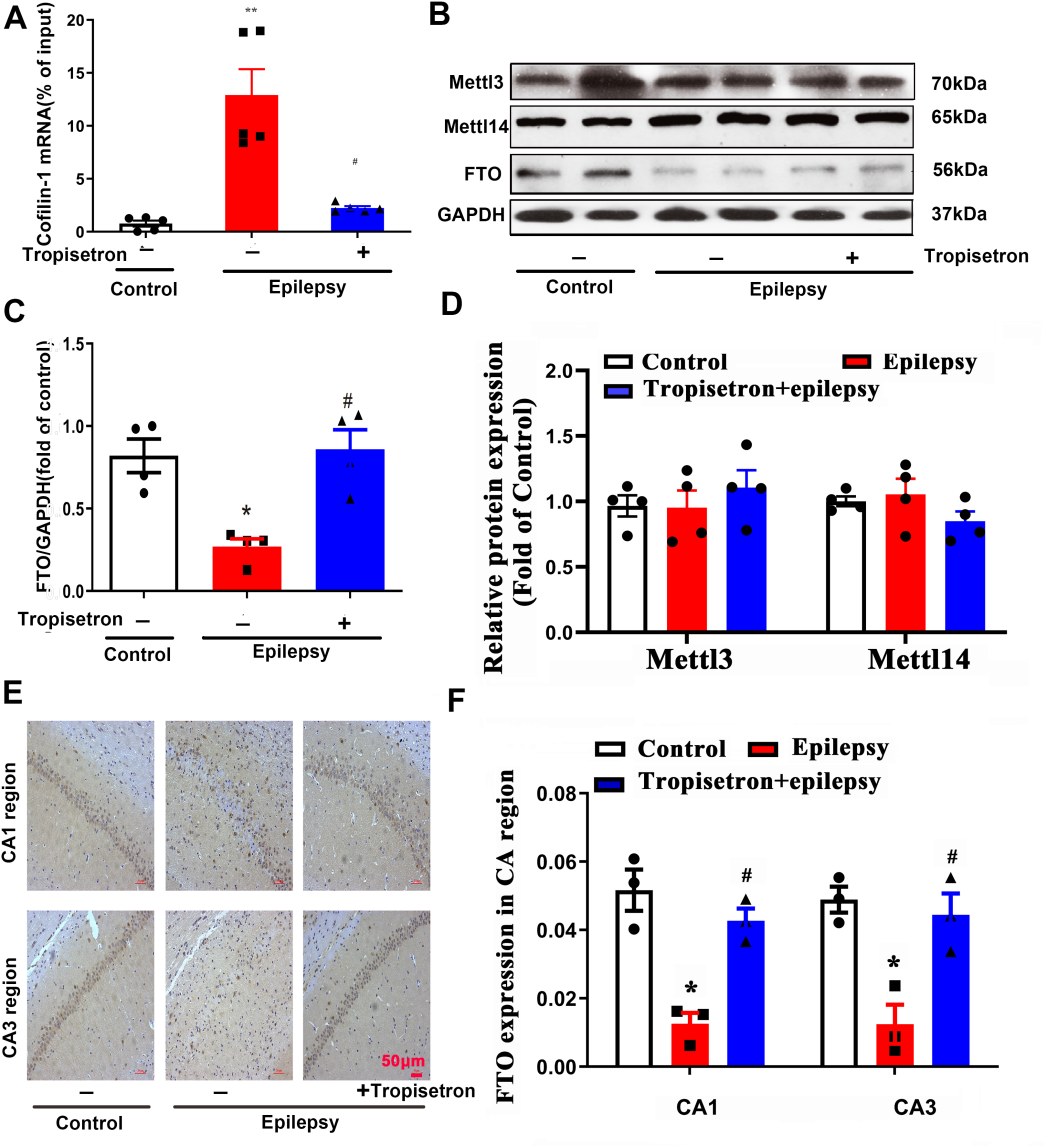
Tropisetron inhibits cofilin‐1 m^6^A RNA methylation in epileptic rats. (A) The cofilin‐1 m^6^A RNA methylation was assessed by MeRIP; **p < 0.01, control versus epilepsy; ^#^ *p* < 0.05 epilepsy versus epilepsy + tropisetron; Mean ± SEM, *n* = 5. (B) The expression of RNA methylation modifying enzyme in hippocampus; (C) Quantitative analysis of FTO; (D) Quantitative analysis of the Mettl3 and Mettl14; *p < 0.05 control versus epilepsy; ^#^ *p* < 0.05 epilepsy versus epilepsy + tropisetron; Mean ± SEM, *n* = 4. (E) The immunohistochemistry analysis of FTO in the hippocampus; (F) Quantitative analysis of FTO; *p < 0.05 control versus epilepsy; ^#^ *p* < 0.05 epilepsy versus epilepsy + tropisetron; Mean ± SEM, *n* = 3.

To explore the underlying mechanisms behind the observed discrepancy between the protein and mRNA expression of cofilin‐1, a comprehensive investigation was conducted. Cofilin‐1 is predicted to have a high number of N^6^‐methyladenosine (m^6^A) modification sites, as indicated by the SRAMP (sequence‐based RNA adenosine methylation site predictor) database (Figure S1). This suggests that cofilin‐1 undergoes m^6^A modification at the RNA level. In our study, methylated RNA immunoprecipitation (MeRIP) analysis revealed a significant increase in m^6^A RNA methylation of cofilin‐1 in epileptic rats compared to control rats (Figure 7A). Importantly, treatment with tropisetron effectively reduced the m^6^A RNA methylation of cofilin‐1 (Figure 7A).

The enzymes involved in m^6^A RNA methylation, including methyltransferase‐like protein 3 and 14 (Mettl3, Mettl14), as well as demethylase, fat mass and obesity‐associated (FTO) were further examined. Our finding revealed that there was no significant change in the expression of Mettl3 and Mettl14 in TLE rats (Figure 7B–D). Furthermore, the administration of tropisetron did not have any influence on the expression of Mettl3 and Mettl14 (Figure 7B–D). In contrast, our study demonstrated a decreased expression of FTO in both hippocampal slices and tissue homogenates of epileptic rats (Figure 7B,C,E,F). This suggests that the demethylation process of RNA is disturbed in epileptic rats. However, tropisetron treatment restored this change (Figure 7B,C,E,F). Based on these findings, it can be inferred that tropisetron may regulate the m^6^A modification of cofilin‐1 by modulating the activity of FTO, thereby restoring the expression of cofilin‐1 in epileptic conditions.

Interestingly, Rhein, an FTO inhibitor [31], prevented tropisetron's action on hippocampal neuron synapses, implying the involvement of FTO in tropisetron's effect on synaptic plasticity (Figure S2).

## 4 Discussion

In the present study, we have demonstrated for the first time that tropisetron, an antiemetic drug used in tumor chemotherapy, can reduce the SRS and alleviate cognitive impairment and HS in TLE rats. The potential mechanisms underlying these effects may involve the reduction of glutamate levels, improvement of cholinergic transmission, anti‐neuroinflammatory properties, and inhibition of synaptic remodeling. Therefore, our findings suggest that tropisetron exhibits both anti‐epileptic and neuroprotective effects in TLE rats.

The imbalance of the glutamatergic‐GABAergic excitatory and inhibitory network plays a pivotal role in epileptogenesis [6]. Our study revealed that tropisetron treatment led to a reduction in glutamate levels by activating α7nAChRs, which likely contributed to the decreased SRS in TLE rats. However, previous study has shown that activation of α7nAChRs enhances glutamate release in hippocampal pyramidal neurons [9]. Notably, the activation of α7nAChRs of astrocytes also plays a role in glutamate clearance by regulating glutamate uptake transporters [32, 33]. In line with this, our research demonstrated that tropisetron upregulated the expression of GLT‐1 via α7nAChRs without affecting NMDA receptors in the hippocampus of TLE rats. Based on these findings, we postulate that tropisetron may influence astrocytes to enhance the clearance of glutamate. It should be noted that no changes in GABA levels were observed following tropisetron administration. The intricate modulation of GABAergic neurons by α7nAChRs remains a complex area [34]. Additionally, the loss of GABAergic neurons in the hippocampus of TLE rats may contribute to disrupted neurotransmitter release.

Cholinergic neurons serve as an important part of the CNS by regulating neuronal excitability, modulating synaptic transmission, and facilitating synaptic plasticity. It has been demonstrated that ACh and cholinergic neurotransmission are involved in epileptogenesis [35]. Our present research indicated that tropisetron improved epileptic‐induced deficits in cholinergic transmission by increasing ACh levels and α7nAChR expression in the hippocampi of TLE rats. Growing studies have found that cholinergic transmission in various brain regions is related to focally induced epilepsy [36, 37]. Several conventional anti‐epileptic drugs, including carbamazepine and zonisamide, have been shown to increase ACh levels by promoting its synthesis and release in distinct brain regions [38, 39]. Hence, the amelioration of cholinergic transmission may represent one of the mechanisms through which tropisetron exerts its anti‐epileptic effects in TLE rats. ACh acts as a neuromodulator in the brain [40]. Thus, we speculated that tropisetron restored the balance between excitation and inhibition by enhancing cholinergic transmission in the brain of TLE rats. Notably, we observed that α‐bgt did not markedly inhibit the effects of tropisetron on ACh levels. This could be due to the potential of chronic α‐bgt treatment to promote the release of ACh in the CNS [41, 42, 43].

Hippocampal neuronal injury and gliosis are involved in the development of TLE, and chronic inflammation aggravates this process [44]. In our present study, tropisetron treatment attenuated hippocampal neuron loss and inhibited gliosis in TLE rats. The loss of hippocampal pyramidal neurons and gliosis primarily contribute to learning and memory impairment in TLE. This neuroprotective effect may explain why tropisetron reduced cognitive deficits in TLE rats. Hippocampus is particularly susceptible to neuroinflammatory insult due to its high density of receptors for inflammatory mediators [45]. α7nAChRs have been identified as key regulators of the “brain cholinergic anti‐inflammatory pathway” [46]. In our study, tropisetron inhibited neuroinflammation by the activation of α7nAChRs, which partly accounts for its neuroprotective effects on the hippocampus. Glucocorticoids (GCs) and its receptors (GR) constitute another endogenous anti‐inflammatory pathway in the brain. In our present study, TLE rats exhibited elevated levels of GCs and reduced expression of GR, while tropisetron treatment upregulated GR expression without altering GCs levels in the hippocampus of TLE rats. Interestingly, α‐bgt did not counteract this effect, suggesting an additional potential mechanism underlying tropisetron's neuroprotection in TLE rats, apart from α7nAChRs‐ mediated action.

Synaptic plasticity is associated with hippocampal hyperexcitability in TLE [47]. Evidence from both animal models and patients has demonstrated that seizure‐induced neuronal loss and alterations in synaptic contacts lead to aberrant neuronal networks, thereby enhancing synaptic efficiency [47]. Hyperexcitable neuronal networks are associated with abnormal dendritic spine formation, which is linked to altered expression of synaptic cytoskeletal proteins [48]. In our study, tropisetron treatment reduced epileptic‐induced aberrant dendritic spines and restored F‐actin expression, suggesting its effect on maintaining synaptic plasticity and stability. CaMKII plays a crucial role in the process of synaptic plasticity, during which CaMKII regulates the structure, functions, and localization of dendritic spines by phosphorylating actin‐binding proteins and influencing the bundling of F‐actin [28, 49, 50]. Tropisetron treatment reduced the elevated expression of CaMKII‐α and II‐β in the hippocampus of TLE rats, and this effect may rescue the aberrant dendritic spine formation by modulating the actin cytoskeleton. Cofilin‐1, a critical actin‐binding protein that translocates into the spine and transiently severs the filaments, is involved in the polymerization/depolymerization of F‐actin [30, 51]. In the present study, tropisetron treatment resulted in an upregulation of the protein expression of cofilin‐1 in the hippocampus of TLE rats. Consequently, we speculate that the inhibition of aberrant dendritic spine formation is linked to the regulation of F‐actin dynamics in TLE following tropisetron treatment.

Interestingly, we noted a discrepancy between the protein and mRNA expression of cofilin‐1 in our study, suggesting the possible involvement of post‐translational modifications in the regulation of cofilin‐1 expression. Further investigation revealed the presence of high m^6^A modification in cofilin‐1 and reduced FTO levels in the hippocampus of TLE rats, with tropisetron treatment reversing this alteration. This suggests a role of epigenetic regulation in anti‐epileptic effect of tropisetron. Although studies have shown that epigenetic regulation is involved in epileptogenesis [52], further investigation is needed to explore the relationship between the anti‐epileptic effect of tropisetron and epigenetics regulation, as well as its potential mechanisms.

Taken together, our present findings demonstrated that α7nAChR activation may represent a crucial mechanism underlying tropisetron's anti‐epileptic effect and neuroprotection through the modulation of glutamate metabolism, enhancement of cholinergic transmission, anti‐neuroinflammation, and regulation of synaptic plasticity. In particular, our study revealed that tropisetron's anti‐epileptic effect involves epigenetic regulation. Tropisetron is a serotonin receptor antagonist that primarily targets the 5‐HT_3_ receptor, except for activating α7nAChR. However, our present study solely concentrates on exploring the contribution of α7nAChRs to tropisetron's antiepileptic effects, as we did not investigate any potential involvement of the 5‐HT_3_ receptor in our experiment. Hence, further studies are required to determine whether 5‐HT_3_ receptor also plays a role in tropisetron's antiepileptic effects and the mechanism of epigenetic regulation. Additionally, the specific molecular mechanisms by which tropisetron interacts with α7nAChRs requires elucidation in future in vitro studies. Although widely used as an antiemetic medicine in clinic, tropisetron has recently shown a promising prospect for treating CNS diseases. Our current study presents significant evidence that tropisetron has the potential for the treatment of epilepsy.

## Author Contributions

M.Z., W.L., X.Q., and X.S. conceived and designed the research. X.Q., X.S., J.D., Z.Y., J.Z., J.Z., T.Z., and S.H. performed experiments and analyzed and interpreted the data. X.L. performed the statistical analysis. X.Q. and X.S. wrote the manuscript. M.Z. revised the manuscript. M.Z. and W.L. supervised the project.

## Conflicts of Interest

The authors declare no conflicts of interest.

## Supporting information


Data S1.

## Data Availability

The raw data and materials that support the findings of the study are accessible from the corresponding author upon reasonable request.
