# Supplementary material for: Tropisetron, an Antiemetic Drug, Exerts an Anti‐Epileptic Effect Through the Activation of α7nAChRs in a Rat Model of Temporal Lobe Epilepsy
Source: CNS Neurosci Ther. 2024 Oct 24;30(10):e70086. doi: 10.1111/cns.70086 (PMC11500210; doi:10.1111/cns.70086)
Supplement: Supplementary file 1 — Data S1. [file CNS-30-e70086-s001.docx]

**Tropisetron, an antiemetic drug, exerts an anti-epileptic effect through the activation of α7nAChRs in a rat model of temporal lobe epilepsy**

Xu Qian^1^, Xinwen Sheng^1,2^, Jiqiang Ding^3^, Zulipiya Yiming^1^, Jingjun Zheng^1^, Jiagui Zhong^3^, Tengyue Zhang^1^, Xuemei Li^1^, Shuqiao He^1^, Wei Li^3^, Mei Zhang^1^

*^1^Department of Clinical Pharmacy, School of Pharmaceutical, Guangzhou Medical University and Key Laboratory of Molecular Target & Clinical Pharmacology, Guangzhou, Guangdong, China,511436, China*

*^2^Department of Pharmacy, the First Affiliated Hospital of Guangzhou Medical University, Guangzhou, Guangdong, China,* *510120, China*

*^3^Department of Neurosurgery, the Six Affiliated Hospital (Dongguan Eastern Central Hospital), Jinan University, Dongguan, Guangdong, 523560, China*

**Correspondence**

Mei Zhang, Department of Clinical Pharmacy, School of Pharmaceutical, Guangzhou Medical University and Key Laboratory of Molecular Target &Clinical Pharmacology, Guangzhou, Guangdong, China,511436, China. E-mail: [zhmeic@gzhmu.edu.cn](mailto:zhmeic@gzhmu.edu.cn).

Wei Li, Department of Neurosurgery, the Six Affiliated Hospital (Dongguan Eastern Central Hospital), Jinan University, Dongguan, Guangdong, 523560, China. E-mail: tliwei@jnu.edu.cn.

**Funding information**

Guangdong Provincial Natural Science Foundation Project, Grant/Award Number: 2023A1515012287; Dongguan Social Development Technology Project Grant/Award Number:20221800905532; Jinan University Medical Union Foundation, Grant/Award Number: YXZY2022023; Guangzhou Medical University student innovation ability enhancement program, Grant/Award Number: 02-408-240603025.

The first two authors contributed equally to this work.

**Highlights**

•Tropisetron reduces spontaneous recurrent seizures in TLE rats.

•Tropisetron improves cognition impairment in TLE rats.

•Tropisetron attenuates hippocampal sclerosis in TLE rats.

•Tropisetron inhibits neuroinflammation in TLE rats.

•Tropisetron represses synaptic reorganization in the hippocampus of TLE rats.

•Tropisetron inhibits the m^6^A methylation modification of synapse-associated proteins cofilin-1 in TLE rats.

**Supplementary materials**

**Figure S1**


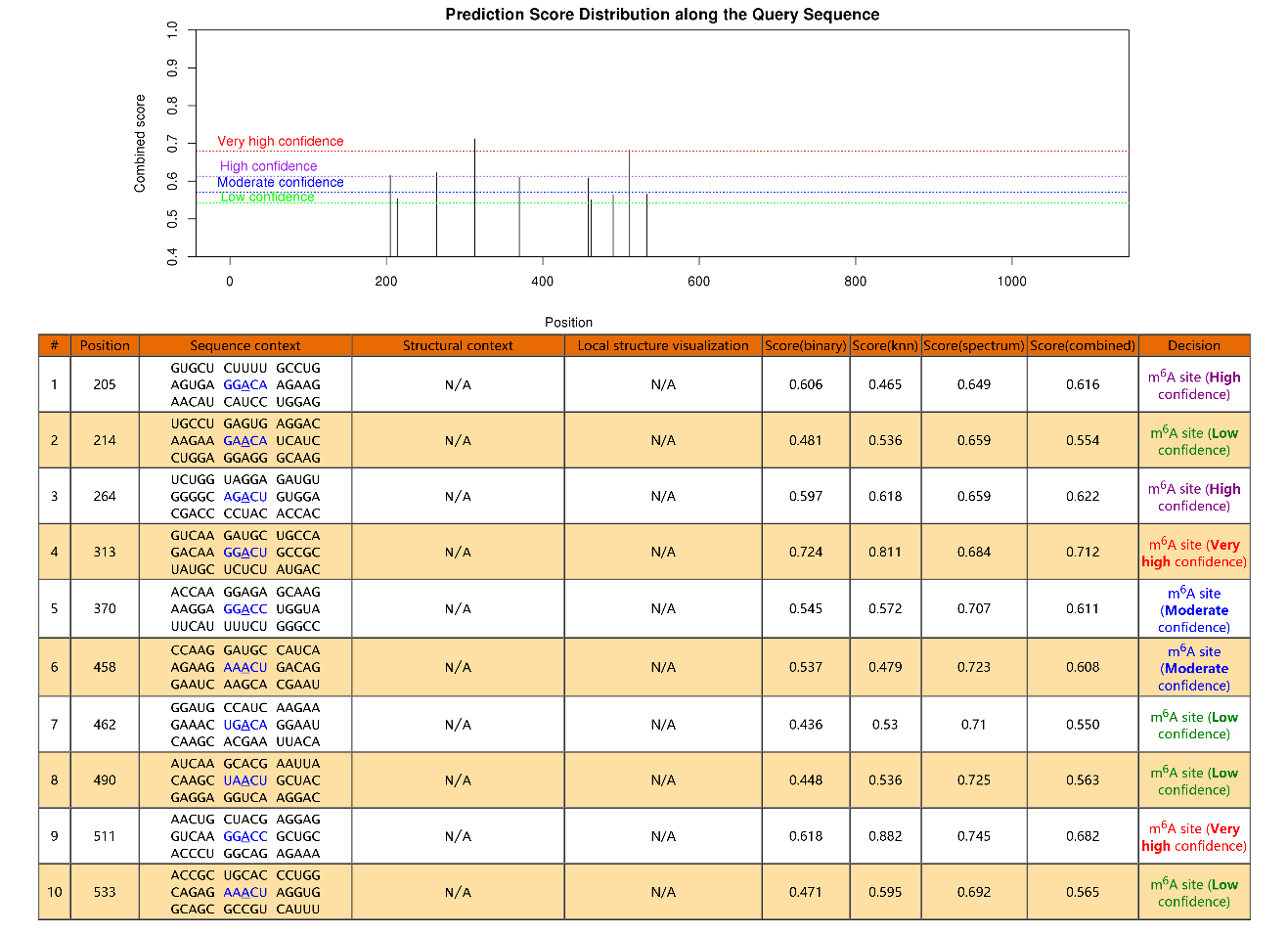


**FIGURE S1** Prediction score distribution of cofilin-1 along the query sequence by the SRAMP.

**Figure S2**


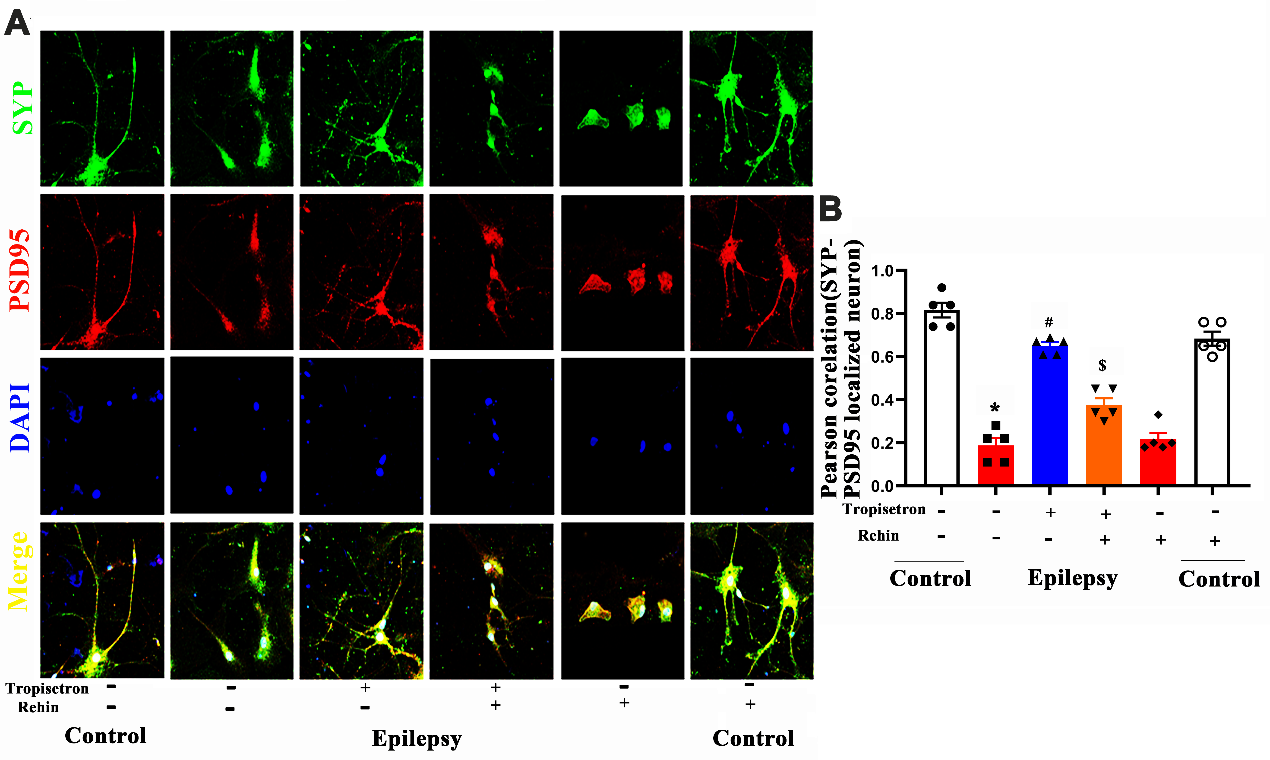


**FIGURE S2** Tropisetron increased hippocampal neuron synapses via FTO in epileptic rats. A: Double-label immunofluorescence analysis of primary hippocampal neuron with SYP (green) and PSD95(red); B: Quantitative analysis of colocalization of neuron and α7nAChRs. ^*^*P* < 0.05 control vs. epilepsy; ^**^*P* < 0.01, control vs. epilepsy; ^#^*P* < 0.05 epilepsy vs. epilepsy + tropisetron; **^##^***P* < 0.01, epilepsy vs. epilepsy + tropisetron; ^$^*P* < 0.05, epilepsy + tropisetron vs. epilepsy + tropisetron + Rhein; Mean±SEM, n=5.

**MATERIALS AND METHODS**

**1. The primary rat hippocampal neurons culture**

The brains were obtained from SPF rat pups aged postnatal day 0–1 and washed with 5 mL of L-15 culture medium to clean the tissue. The hippocampus was then isolated and placed in a culture dish containing DMEM culture medium, where it was finely sheared into 0.5-1mm^2^ pieces. The dissected tissue was then incubated in 0.25% trypsin at 37°C for 6 minutes. After termination of digestion with 1 mL FBS, the mixture was centrifuged at 800 rpm for 5 minutes at room temperature. The supernatant was removed, and the pellets were gently resuspended in culture medium. Next, the cell suspensions were filtered through a 70 μm strainer and added onto air-dried PDL-coated coverslips at a density of 5–7×10^5^ cells/well for approximately 10 hours. Subsequently, the DMEM was replaced with pre-warmed Neurobasal-A culture medium at 37°C. The dissociated culture was replenished by adding fresh Neurobasal-A culture medium to each well every three days thereafter.

**2. Establish the Sombati's epileptic cell model and drug treatment**

The primary rat hippocampal neurons were cultured for 10 days, after which the medium was replaced with Mg^2+^-free medium for a duration of 3 hours to induce the Sombati's cell model. Subsequently, the primary rat hippocampal neurons were pre-treated with tropisetron (100nM) or rhein (50μM) for 24 hours, followed by a change in the medium to Mg^2+^-free medium for an additional 3 hours.

**3.Immunofluorescence analysis of the primary rat hippocampal neurons**

The cell crawling slides were prepared as described above for the culture of primary rat hippocampal neurons. Round coverslips were incubated overnight with primary antibodies (anti-SYP and anti-NeuN mouse mAb, 1:100, CST; anti-PSD95 rabbit mAb, 1:100, CST) at 4°C. After washing, the slices were incubated with IgG-alexa fluor-555 (goat anti-mouse, 1:1000, CST) and IgG-alexa fluor-647 (goat anti-rabbit, 1:1000, CST) in the dark for 1 hour. Subsequently, the DAPI dye solution (CST) was used to stain the nucleus for 10 minutes. The slices were observed with a confocal microscope, and images of the coverslip were obtained at 60× magnification.
